# Signals for antigen-independent differentiation of memory CD8^+^ T cells

**DOI:** 10.1007/s00018-021-03912-9

**Published:** 2021-08-16

**Authors:** Eliza Mari Kwesi-Maliepaard, Heinz Jacobs, Fred van Leeuwen

**Affiliations:** 1 Division of Gene Regulation, Netherlands Cancer Institute, 1066CX Amsterdam, The Netherlands; 2 Division of Tumor Biology and Immunology, Netherlands Cancer Institute, 1066CX Amsterdam, The Netherlands; 3 Department of Medical Biology, Amsterdam UMC, University of Amsterdam, 1105AZ Amsterdam, The Netherlands

**Keywords:** CD8^+^ T cell, Antigen-inexperienced memory, Virtual memory, Innate memory

## Abstract

Conventional CD8^+^ memory T cells develop upon stimulation with foreign antigen and provide increased protection upon re-challenge. Over the past two decades, new subsets of CD8^+^ T cells have been identified that acquire memory features independently of antigen exposure. These antigen-inexperienced memory T cells (T_AIM_) are described under several names including innate memory, virtual memory, and ﻿memory phenotype. ﻿T_AIM_ cells exhibit characteristics of conventional or true memory cells, including antigen-specific responses. In addition, they show responsiveness to innate stimuli and have been suggested to provide additional levels of protection toward infections and cancer. Here, we discuss the current understanding of T_AIM_ cells, focusing on extrinsic and intrinsic molecular conditions that favor their development, their molecular definitions and immunological properties, as well as their transcriptional and epigenetic regulation.

## Introduction

T lymphocytes (T cells) are key players in cellular immune responses. Each T cell is equipped with a unique, clonotypic T cell receptor (TCR) capable of recognizing a number of small protein fragments (peptides) in complex with MHC molecules (pMHC) that are displayed on the surface of antigen-presenting cells. This ensures that, on a population level, T cells can respond to almost any infection. After a primary T cell-mediated immune response, a subset of antigen-specific T cells persists as long-lived memory T cells in lymphoid organs. Upon antigen re-challenge, memory cells are pre-programmed to differentiate rapidly into effector cells warranting effective secondary immune responses [1, 2].

In addition to these ‘true’ memory T cells (T_TM_), other, non-conventional memory T cells are being identified and characterized, as documented by an increasing number of independent studies. Specifically, CD8^+^ T cells with a memory phenotype can arise in an antigen-inexperienced manner, i.e., without previously being activated [3–5]. For simplicity, we here refer to these cells as antigen-inexperienced memory T cells (T_AIM_). T_AIM_ cells constitute around 10–40% of total peripheral CD8^+^ T cells, indicating their potential importance in the immune system [6–13]. Two major CD8^+^ T_AIM_ cell types have been described: innate memory and virtual memory cells. Innate memory (T_IM_) cells arise in the thymus, where they mostly depend on IL-4 [5, 14]. Virtual memory (T_VM_) cells develop in the periphery and depend on IL-15 [15]. However, the distinction between and origin of the two types of T_AIM_ cells is still under debate [3, 16].

Functionally, T_AIM_ cells partially resemble conventional antigen-experienced T_TM_ cells by mounting a rapid immune response upon antigen stimulation [3, 5]. The immune response of T_AIM_ cells from young animals is particularly fast, suggesting that they provide extra protection in young animals when the immune system is not fully established [17]. In aged mice, T_AIM_ cells are major contributors to the anti-influenza response [18]. Besides mediating a TCR-dependent immune response, T_AIM_ cells have also been shown to provide antigen non-specific bystander protection in response to innate stimuli [8, 9, 19]. In addition, a potential role for T_AIM_ cells in the anti-tumor response has been suggested: T_AIM_ cells are able to infiltrate tumors and express high densities of the inhibitory receptor PD-1, a common target for immunotherapy [12, 20]. Furthermore, chemotherapeutic treatment of tumor cells increases the abundance of T_AIM_ cells [21]. These T_AIM_ cells can inhibit tumor growth in an MHC-I independent manner [21]. This unique responsiveness of T_AIM_ cells to different stimuli in combination with the substantial number of T_AIM_ cells warrants detailed investigation into this ‘neglected’ CD8^+^ T cell compartment.

Another type of memory-phenotype cells are those induced by homeostatic proliferation in a lymphopenic environment [22]. It has been suggested that under lymphopenic conditions, these homeostatic proliferation-induced memory-phenotype cells (T_HP_) arise from naïve T cells exposed to high levels of the homeostatic cytokine IL-7 [22]. The sum of these insights reveals that the T cell memory pool is very heterogenous and that the acquisition of T-cell memory features does not follow a single trajectory. Increasing our knowledge about T_AIM_ cells will help to better understand the role of these diverse memory T cell subsets in controlling specific immune responses. In this review we will not discuss T_HP_ (for a detailed review see Sprent et al. [22]), but focus on T_AIM_ cells that arise independent of experimentally induced lymphopenia. This review focuses on the origin of T_AIM_ cells and how their numbers and functions are regulated under specific physiological and immunological conditions. Furthermore, we will discuss transcriptional and (epi)-genetic regulation underlying the generation of T_AIM_ cells.

## Markers of T_AIM_ cells

How are CD8^+^ T_AIM_ cells identified and distinguished from T_TM_ cells? Several definitions of T_AIM_, T_VM_ and T_IM_ are being used in the literature, defining the subsets based on surface markers, functionality and antigen-specificity [3–5]. The basic definition of a T_AIM_ cell is a T cell that has not been exposed to foreign antigen but has memory-like features. This antigen-inexperienced nature of T_AIM_ cells can be defined in several ways. One option is tetramer staining to identify CD8^+^ T cells that are specific for defined pMHC class I complexes they have not been challenged with (e.g., the chicken ovalbumin OVA and vaccinia virus B8R epitopes) [9, 13, 15, 23–25]. Accordingly, one expects that CD8^+^ T cells capable of binding the OVA and B8R tetramers are naïve, since they have not been previously challenged with the same pMHCs. Even though a single TCR can detect many different pMHCs, the presence of memory-phenotype cells among these tetramer-binding T cells in unchallenged mice indicates that these cells are not T_TM_ cells, but instead represent T_AIM_ cells. Another option to guarantee that cells are antigen inexperienced is to use CD8^+^ T cells with a transgenic (tg) TCR, ideally on a background that prevents expression of endogenous TCRs, such as a RAG1/2 knock-out background [9, 25, 26]. When all CD8^+^ T cells express the same tgTCR that only recognizes a synthetic peptide (e.g., the OT-I TCR recognizing the SIINFEKL epitope derived from OVA), the memory-phenotype cells identified without exposure to that specific antigen are T_AIM_ cells. Exposing the cells to the specific synthetic antigen will result in the formation of T_TM_ cells. To identify T_AIM_-specific markers T_AIM_ cells have been compared to naïve (T_N_) and T_TM_ cells. These markers are now broadly applied to determine whether memory-phenotype cells in unchallenged mice are T_AIM_ cells. Awareness of the T_AIM_ subset and the specific markers to detect them is important to determine whether memory-phenotype cells in unchallenged mice are T_TM_ or T_AIM_. Unchallenged mice housed under specific pathogen-free (SPF) conditions typically have a memory-phenotype population, of which the majority (80–90%) are T_AIM_ cells [6, 27, 28]. Many studies in the past did not use T_AIM_-specific markers and as a result were regarded all as memory-phenotype cells, including T_AIM_, as T_TM_. The current knowledge about the heterogeneity of the memory-phenotype population in unchallenged mice warrants a re-evaluation of our understanding of conventional T-cell differentiation [4]. Precise identification of the different memory subsets is thus required to gain further insight into the heterogenous process of memory T cell differentiation.

T_AIM_ cells are generally defined by several combinations of surface markers. They express many of the surface markers of conventional or true memory cells, including CD44 and CD62L. However, they can be distinguished from T_TM_ cells by low expression of CD49d, a marker of previous antigen experience [13, 15], high surface levels of CD122, part of the IL-2 and IL-15 receptor [13, 15, 29] and increased surface levels of CD5 [9, 30]. Furthermore, NKG2D, an activating cell surface receptor, is upregulated on a subset of T_VM_ cells, but not on all IL-4-induced T_AIM_ cells [9, 24, 31]. Besides these surface markers, the intracellular expression of the transcription factors T-bet and EOMES is also used to distinguish T_AIM_ from conventional memory cells. In C57Bl/6 mice, T_VM_ cells express high levels of both T-bet and EOMES, whereas T_TM_ are either T-bet^+^ or EOMES^+^ depending on being effector memory or central memory, respectively [26]. In BALB/c mice, T_IM_ cells both in thymus and periphery are T-bet^−^EOMES^+^ [14, 32]. In the thymus, T_IM_ cells are often described as CD44^+^CD122^+^EOMES^+^ CD8^+^CD4^−^ thymocytes that express the chemokine receptor CXCR3 [14, 33] and have reduced levels of CD24, a feature of more mature CD8^+^ thymocytes [34]. A subset of the peripheral T_AIM_ population also expresses CXCR3 [35]. Based on CD44 and CXCR3 expression, CD8^+^ T cells can be divided into naïve (T_N_; CD44^−^CXCR3^−^), intermediate (CD44^+^CXCR3^−^), and memory phenotype (CD44^+^CXCR3^+^) [31, 32]. Whether T_IM_ and T_VM_ are different stages of one T_AIM_ subset or different subsets is still the topic of ongoing discussion [4, 16, 36]. It has recently been proposed that CD122 expression can function as marker to distinguish T_IM_ and T_VM_ in the periphery, with T_IM_ cells having lower expression of CD122 than T_VM_ but higher than T_N_ [11]. A detailed overview of T_AIM_ surface markers is provided in Table 1.

**Table 1:** Markers of T_AIM_ cells

Marker	Function	Subset	Reference
CD122^high^	Part of the IL-2 and IL-15 receptor [29]. Expression of CD122 is induced by EOMES and T-bet [37]. Lower CD122 expression in T_IM_ compared to T_VM_ [11].	T_IM_ and T_VM_	[13, 15]
CD49d^low^	α4β-Integrin; Part of the very late antigen-4 (VLA4) integrin that is involved in homing of effector T cells to the site of infection. CD49d is highly expressed on antigen-activated effector memory (T_EM_) and effector (T_EFF_) CD8^+^ T cells [38]. CD49d upregulation is suppressed by IL-4 [38].	T_IM_ and T_VM_	[13, 15]
CD5^high^	Negative regulator of TCR signaling [39–41]. CD5 surface levels function as a proxy for TCR-binding affinity of the thymus [39–41].	T_VM_	[9, 30]
NKG2D^low/high^	Natural killer group 2 member D is a receptor expressed on activated CD8 T cells in mice and constitutively on CD8 T cell in human [42]. It functions as a co-stimulatory receptor [42]. NKG2D expression can be downregulated by IL-4 [31].	High in a subset of T_VM_	[9, 24, 31]
CXCR3^high^	Chemokine receptor involved in migration of T cells to peripheral site of infection [43].	T_IM_ and T_VM_	[14, 31–33, 35]

## Mechanisms inducing differentiation toward T_AIM_

The precise mechanisms that induce differentiation toward innate and virtual memory development are not completely understood, but two major components have been identified: quality of the TCR signal and cytokine signaling in the developmental niche.

### TCR signaling in T_AIM_ cell differentiation

The quality of the TCR signal critically contributes to the decision making of developing and differentiating T cells. During thymic maturation, thymocytes expressing a TCR with very weak or strong affinity for self-peptides die by neglect or negative selection, respectively, and only thymocytes expressing intermediate affinity TCR are positively selected and migrate into the periphery [44]. In the periphery, T cells require tonic TCR signals for their survival [22]. Strong binding of an antigen to the TCR results in activation of the peripheral T cell and differentiation toward memory and effector cells [45, 46], but only when critical co-stimulatory signals are provided; otherwise the cells will become anergic, a long-lasting stage of T cell non-responsiveness [47]. The strength of TCR signaling in the thymus and periphery thus determines the fate of a T cell.

One characteristic of T_VM_ cells is their increased CD5 surface level [9, 30]. High CD5 serves as a proxy for high TCR affinity during thymic selection and negatively regulates TCR signaling in the thymus, thereby increasing the positive selection window by preventing negative selection [39–41]. The relative level of CD5 correlates with the percentage of T_VM_ cells in different mouse models [30]. Furthermore, CD5^high^ naïve cells acquire a T_VM_ phenotype more often after adoptive cell transfer than CD5^low^ naïve cells [9]. This suggests that cells with higher affinity for self-antigens or with higher TCR signaling are more prone to become T_AIM_. This is corroborated in a mouse model with increased binding of the tyrosine kinase Lck to the CD8 co-receptor resulting in supraphysiological TCR signaling: mice with this increased TCR signaling have an increased number of T_VM_ [30]. The importance of signaling through the CD8 co-receptor is further demonstrated in a mouse model with constitutive expression of CD8αβ. This results in increased numbers of T_IM_ cells in the thymus, partly caused by extrinsic factors involving other thymic cell subsets and partly through cell-intrinsic mechanism [48]. Furthermore, mice deficient for DOCK2, which contributes to cellular signaling events by activating small G proteins, are more sensitive to TCR stimulation with low-affinity antigens and have increased numbers of T_VM_ cells [10]. This also suggests that increased sensitivity for tonic/self-peptide signaling induces T_AIM_ differentiation. A role for TCR quality was further supported by a study using mice with a transgenic TCRα and TCRβ chain. When these tgTCR mice are bred on a *Rag*-KO background, recombination of endogenous TCR genes is prevented and all T cells express the exact same TCR (OT-I) with the same affinity. Under these Rag-deficient conditions, the percentage of virtual memory cells is low (< 5%) and remains low during aging [25]. However, CD8^+^ T cells from mice on a Rag-proficient background, which are able to recombine the endogenous TCRα chain, generated higher T_VM_ cells at higher frequency and number, both of which increased with age (> 50%) [25]. This indicates that some TCRs are more likely to support the gain of a T_AIM_ phenotype than others.

The TCR repertoire of unchallenged T_AIM_ cells is distinct from that of naïve CD8^+^ T cells (T_N_) [12, 30]. Several TCRs have been identified as T_AIM_-TCRs or T_N_-TCRs, both in mice with an endogenous TCR and in mice expressing a transgenic TCRβ chain [12, 30]. Retroviral expression of transgenic T_AIM_-TCRs contributes to an increased number of T_AIM_ cells in vivo [12, 30]. Cells expressing a T_AIM_-TCR also respond stronger to stimulation with self-peptides by MHC-I on dendritic cells [12]. Furthermore, cells with a T_AIM_-TCR are already EOMES^+^ in the thymus and have increased surface CD5 expression, indicating that TCR-induced T_AIM_ differentiation already starts in the thymus [12, 20]. This increased TCR affinity also correlates with the higher convergence score reported for memory-phenotype (CD44^+^CD122^+^) cells [10]. The convergence score is the number of unique TCR sequences that encode for the same TCR and a higher score is indicative of selection by self-antigens [10]. Interestingly, co-culture of naïve CD8^+^ T cells with chemotherapeutically treated tumor cells results in an increase of T_VM_-phenotype cells independent of MHC-I [21], suggesting that other signaling pathways might overrule TCR signaling in certain conditions. Taken together, this data suggests a model in which T_AIM_ cells arise as a result of an intrinsically higher TCR signaling potential.

Given the increased TCR signaling in T_AIM_ cells, Drobek et al. studied if T_AIM_ cells are more likely to respond strongly to self-peptides and induce auto-immune pathology [30]. They found that T_AIM_ cells do not break self-tolerance and are less effective in inducing auto-immune pathology compared to T_TM_ cells [30]. This suggests that T_AIM_ differentiation might function as an escape mechanism for self-reactive CD8^+^ T cells to avoid auto-immunity [30, 49]. Alternatively, when T_AIM_ cells recognize self-peptides, they might ‘not properly’ be activated/primed and as a result of this have reduced cytotoxic capacities compared to properly activated T_TM_ cells. In light of this, it is relevant to note that regulatory CD8^+^ T cells also have a CD122^+^CD49d^−^ memory phenotype [50], although to our knowledge the immune-regulatory function of T_AIM_ cells has not been studied.

### Cytokine signaling in T_AIM_ cell differentiation

Cytokine signaling is critical to ensure that activated T cells follow the right trajectory of differentiation. The two main cytokines involved in T_AIM_ development are IL-4 for T_IM_ and IL-15 for T_VM_ cells. IL-4 is mainly produced by invariant NKT (iNKT) and γδ-T cells in the thymus [14], although other thymic populations have also been reported as a source for IL-4 [51]. Mouse models in which the number of IL-4-producing iNKT cells, γδ-T cells or CD4 single positive T cells is increased have more innate memory cells [14, 52–55] as reviewed in [56]. Furthermore, the number of iNKT cells is higher in BALB/c mice compared to C57Bl/6 mice, correlating with a higher number of T_IM_ cells [14, 57–59]. When IL-4 signaling is reduced in mouse models with increased IL-4 production, either by ablating iNKT cells, deleting the IL-4 receptor or by blocking IL-4 with antibodies, the T_IM_ population is diminished, indicating that T_IM_ cells require IL-4 [53, 60]. On the other hand, in vivo treatment with IL-4/anti-IL4 antibody complex results in increased number of CXCR3^+^ CD8^+^ T cells that have increased EOMES expression, indicating that increased IL-4 levels contribute to T_IM_ differentiation [34, 61]. The exact mechanism of how increased IL-4 signaling stimulates T_IM_ differentiation is not known. It has been reported that IL-4 drives EOMES expression through Stat6- and AKT-dependent pathways [32, 62]. However, T_IM_ cells do not depend on increased IL-4 production under all conditions. There are also reports of T_IM_ cells arising independently of excess IL-4 production [63]. Taken together, IL-4 is important, and in most cases, required for T_IM_ differentiation in the thymus.

Although increased levels of IL-4 can contribute to an increased percentage and number of T_VM_ in the periphery [19, 23, 57, 64], IL-15 is the most important cytokine for peripheral T_AIM_ cells [3]. IL-15 is a key driver of homeostatic CD8^+^ T cell proliferation under steady-state conditions and regulates memory effector functions [65]. Several lines of evidence reveal that in the periphery, T_AIM_ cells depend on IL-15. First, ablation of IL-15 production or abrogation of IL-15 signaling completely inhibits T_AIM_ differentiation and survival [9, 15, 66, 67]. Second, T_AIM_ differentiation in IL-15 depleted mice can be rescued by injecting IL-15/Rα complexes [9, 15]. Third, CD122, part of the IL-15 receptor complex, is more highly expressed on T_VM_ compared to naïve and T_TM_ [15]. Furthermore, a recent study suggests that regulatory CD4 T cells regulate T_VM_ differentiation by inhibiting IL-15 trans-presentation by CD11b^+^ dendritic cells (DCs) [68]. How increased IL-15 signaling leads to the formation of T_AIM_ cells has not been fully described yet. It has been suggested that IL-15 signaling results in homeostatic expansion of T_VM_ cells that are already present in the periphery [15]. However, IL-15 signaling might also contribute toward T_AIM_ differentiation itself. The source of IL-15 for T_AIM_ cells are CD8α + DCs in peripheral lymphoid tissues [15]. This suggests that peripheral T_AIM_ mostly depend on naturally occurring IL-15.

### T_AIM_ cell differentiation: a delicate balance of multiple signals

**Fig. 1:**
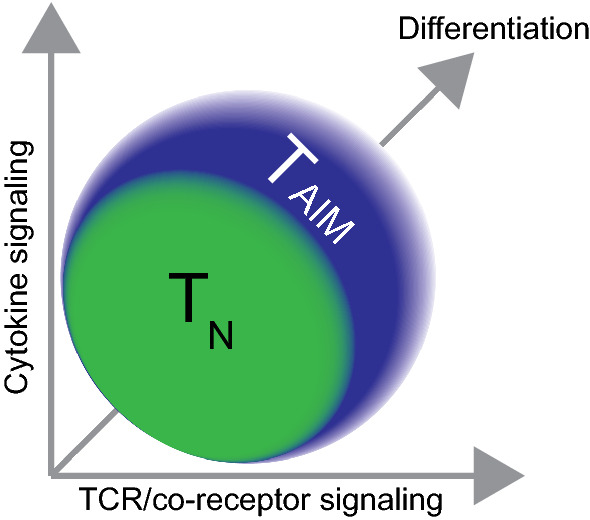
Antigen-inexperienced memory T cell (T_AIM_) differentiation: A delicate balance of integrated signaling. The quality of TCR/co-receptor and cytokine signals are central to T_AIM_ differentiation. Cytokines most important for T_AIM_ cells are IL-4 in the thymus and IL-15 in the periphery. Ablation of these cytokines or their receptors leads to a strong reduction in the number of T_AIM_ cells. The balance between cytokine and TCR signals determines whether a CD8^+^ T cell remains naïve (T_N_) or differentiates into a T_AIM_ cell. The TCR and cytokine signals lead to altered transcriptional output, which is further modulated by epigenetic mechanisms. The extracellular signals, and fluctuations in expression of genes involved in these signaling pathways due to transcription dynamics, as well as differentiation and proliferation together affect T_AIM_ differentiation. The net result is a dynamic population of T_AIM_ cells in mouse and humans

Taken together, the current understanding of signals involved in T_AIM_ differentiation suggests a model in which T_AIM_ differentiation is steered by a very delicate balance of at least two external signals mediated by the TCR and cytokine receptors and integrated intracellularly (Fig. 1). These signals lead to altered transcriptional output, which is further modulated by epigenetic mechanisms (see below). Natural fluctuations in gene expression of key genes that regulate TCR signaling or cytokine signaling or their downstream targets may render cells more or less susceptible to become T_AIM_ cells. This is in line with the current notion that fluctuations in gene expression occur over time within cell populations and that these fluctuations can have long term impact on features of the original cell and its offspring [69, 70]. Among the genes whose fluctuation might affect T_AIM_ differentiation is CD5, a negative regulator of TCR signaling. Fluctuations in CD5 expression may render cells more sensitive to tonic signals provided by self-peptide MHC complexes, making these clones more prone to inappropriate priming and differentiation, especially under pro-proliferative conditions like lymphopenia. In this context, the tyrosine kinase ITK, a mediator of TCR signaling, is also of interest. ITK plays an important role in T_AIM_ differentiation. ITK ablation in the T cell lineage results in increased numbers of T_AIM_ cells [66]. This skewed differentiation is IL-4 dependent and requires an external source of IL-4 [51, 56]. However, Huan and colleges suggest a cell-intrinsic role for ITK in T_AIM_ differentiation [51]. While in vitro TCR activation in combination with IL-4 results in reduced expression of the IL-4 receptor (IL-4R) and EOMES and a decrease in the percentage of T_AIM_ cells in WT CD8 single positive thymocytes, ITK-ablated CD8 single positive thymocytes are less sensitive to TCR activation and maintain higher levels of IL-4R and EOMES and a higher percentage of T_AIM_ cells [51]. This suggests that ITK tunes the response to IL-4 signaling cell intrinsically [51]. Given the role of ITK as a mediator of TCR signaling, TCR signaling might no longer lead to reduced IL-4 receptor expression on CD8 single positive thymocytes in the absence of ITK, thereby rendering the cells more sensitive to IL-4. Cross talk between TCR signaling and cytokine signaling is also shown by the effect of TCR signaling on IL-4-induced expression of EOMES in vivo, where IL-4-induced upregulation of EOMES is stronger upon weak TCR signaling compared to strong TCR signaling [62]. This all suggests a model in which a delicate balance of TCR signaling and cytokine signaling is required to keep cells in a naïve state or induce T_AIM_ differentiation.

## Physiological regulation of T_AIM_ during development and infection

T_AIM_ cells are observed in unchallenged wild-type mice and also in humans. Increased numbers of T_AIM_ cells have been described in several genetically engineered mouse models (GEMM). The number of T_AIM_ cells is also regulated during normal physiological development and it changes during life. Most studies on T_AIM_ are performed in adult mice, which have a small but substantial population of T_AIM_ cells (~ 20% of total CD8^+^ T cells) [6–13]. In young (infant) mice, there is a larger fraction of T_AIM_ cells (~ 35%) [23]. Using an elegant approach to time stamp T cells produced at different ages, Smith et al. mapped the fate of cells from different developmental origins [17]. They showed that in adult mice the CD8^+^ T cells with a fetal origin have around five times more T_VM_ cells than CD8^+^ T cells with an adult origin. This increased T_VM_ frequency in CD8^+^ T cells with a fetal origin is not caused by lymphopenia, but rather is an intrinsic feature of CD8^+^ T cells of early developmental origin [17]. Physiologically, the early-developed cells contribute more rapidly to an immune response compared to late-developed T cells, as shown by a rapidly increased number of early time-stamped cells at the beginning of the immune response. Early-developed T_VM_ cells respond in a more innate-like manner and differentiate predominantly toward short-lived effector cells, whereas late-developed T_VM_ cells give preferentially rise to long-lived memory cells [17]. This suggests that the T_VM_ cells developed during early stages of development might give increased rapid innate-like protection that is needed in young animals with a developing immune system. Importantly, this data shows that the T_AIM_ pool is not homogenous, but that T_AIM_ cells developed during different stages are differentially programmed and have distinct functions.

In aged mice, the percentage and absolute number of T_AIM_ is again increased [9, 11, 28, 71]. The precise mechanism that contributes to this increase is unknown, but also in older individuals T_AIM_ cells depend on a combination of TCR and cytokine signaling. Aged mice that have a transgenic TCR but that are also able to undergo recombination of the endogenous TCRα have increased numbers of T_AIM_, similar to younger mice [25]. This again demonstrates that the differentiation of T_AIM_ cells requires a specific level of TCR signaling. Furthermore, the surface levels of CD5 are increased in unchallenged CD8^+^ T cells in aged mice [71], suggesting a selective advantage due to increased sensitivity for self-antigens. Consequently, in aged mice CD8^+^ T cells expressing TCRs with high affinity for self-pMHC and strong signaling potential increase, favor T_AIM_ differentiation and with time T_AIM_ cells outgrow other CD8^+^ T cells. Similar to T_AIM_ in young mice, T_AIM_ cells in aged mice still depend on IL-15, as was demonstrated by the absence of T_VM_ in aged IL-15 KO mice [6]. Furthermore, expression of CD122, part of the IL-15 receptor, is increased on a subset of T_AIM_ cells in aged BALB/c mice compared to young mice [11]. Taken together, this suggests that T_AIM_ cells that accumulate in older animals require similar TCR and cytokine signals as early-developed T_AIM_ cells.

Functionally, T_AIM_ cells are major contributors to an immune response against influenza in aged mice and gain a T_CM_ CD49d^+^ phenotype afterward [18]. However, when aged T_VM_ and naïve CD8^+^ T cells are transferred together, the T_VM_ cells are outperformed by the naïve cells [18]. Furthermore, aged T_VM_ are more monofunctional, secreting only one effector cytokine, whereas naïve cells are more polyfunctional, secreting several different cytokines [7]. Aged T_VM_ also have a reduced proliferative capacity [25] and they have a senescent phenotype characterized by resistance to apoptosis, increased γH2Ax indicating accumulated DNA damage and increased expression of Bcl-2 family members that control apoptosis [7]. The overall impaired immune response in aged individuals might among other changes also relate to reduced numbers of naïve CD8^+^ T cells and increased numbers of later developed T_AIM_ cells.

There are several indications that the number of T_AIM_ cells is also actively regulated during specific immunological challenges. During helminth infection, the concentration of IL-4 in the thymus increases and this correlates with an increased T_VM_ pool [8, 19]. These newly expanded T_VM_ cells are able to mount a more effective immune response against lytic gammaherpesvirus infection [8] and bacteria [19]. Infection with trypanosomes results in cellular composition changes in the thymus, leading to increased production of IL-4 and IL-15 and an increased percentage of memory-phenotype cells in the thymus although these cells are CD49d^+^ [72]. These T_IM_-phenotype cells offer increased protection against trypanosome infection in an antigen-independent manner [72]. T_IM_-phenotype cells (CD44^+^CXCR3^+^CD49d^+^) are also seen after treatment with metformin, a drug used in treatment of type 2 diabetes, and they can contribute to increased protection against infection with *M. Tuberculosis* [73]. However, the origin of these metformin-induced T_IM_-phenotype cells is not known [73]. Different infections can thus affect the immunological environment and thereby impact T_AIM_ differentiation. Increased numbers of T_AIM_ cells during certain immunological challenges are also seen in humans (discussed later); however, the exact mechanism is not always known. Given this recurrent correlation between infections and T_AIM_ development, further research into how infections promote the generation of T_AIM_ and the potential advantage of this increased T_AIM_ population in establishing immunity is crucial.

Most studies on T_AIM_ cells have been performed in mice housed under SPF conditions. This raises the question whether increased or decreased pathogen exposure affects T_AIM_ differentiation. Several studies have shown that T_AIM_ cells are also present in mice housed under germ-free conditions in a similar frequency as in SPF mice [11–13]. The presence of T_AIM_ cells in ‘dirty’ feral mice has only recently been investigated. Feral mice have an increased frequency of T_AIM_ cells compared to laboratory mice [11]. The frequency of T_AIM_ cells in feral mice is even higher than in F1 offspring of feral mice born in captivity [11]. This indicates that environmental factors may be involved in T_AIM_ differentiation, but the exact factors are not known. Co-housing of feral animals with laboratory animals affects the microbiome of the laboratory animals, but has very limited effect on T_AIM_ cells [11]. Further studies are thus required to determine which environmental factors might impact T_AIM_ differentiation.

Altogether, the number of T_AIM_ cells is not static but physiologically regulated. Both the age at which T_AIM_ cells are formed and the immunological challenges they encounter determine their number and function.

## Transcriptional and epigenetic regulation of T_AIM_ differentiation

To better understand how T_AIM_ cells are regulated, we first need to define their developmental state. Are T_AIM_ cells fully differentiated memory T cells or do they represent a semi-differentiated subset? To answer this question, several studies have looked beyond the small number of surface markers detected by flow cytometry and determined the full transcriptome of T_AIM_ cells. Drobek et al. compared RNA-seq expression profiles of T_N_ (CD44^−^CD62L^+^), T_VM_ (CD44^+^CD62L^+^CD49d^−^) and T_TM_ (K^b^-OVA^+^CD44^+^CD62L^+^, after infection with *Listeria monocytogenes*-OVA) [30]. Based on publicly available datasets of T_N_ and T_TM_ they defined naïve and memory gene signatures. T_VM_ cells are more enriched for memory signature genes than T_N_, but less than T_TM_. For naïve gene signatures T_VM_ are also in between T_N_ and T_TM_. A similar intermediate phenotype is seen for expression of cytokines and chemokines [30]. This suggests that T_AIM_ cells indeed have an intermediate T_N_→T_TM_ differentiation phenotype. However, this does not mean that they are just T_N_ cells that are only differentiated halfway. When compared with T_N_ and T_TM_, T_VM_ cells have their own unique transcriptome features [7, 30]. This is shown by T_VM_ cells clustering away from T_N_ an T_TM_ based on principal component analysis or multi-dimensional scaling analysis [7, 30]. Taken together, T_AIM_ cells are a unique T cell subset with an intermediate differentiation phenotype as well as a unique transcriptome.

Further comparison of T_N_ and T_VM_ shows that T_VM_ cells have high expression of genes related to cell killing, inflammatory cytokines, granzymes, and genes related to NK cell receptors, and reduced expression of CD49d [7]. Furthermore T_VM_ cells have increased expression of genes involved in cytokine sensing, including IL-15 signaling mediators, and of the transcription factor EOMES [36]. A similar trend is seen for thymic T_IM_ cells, which are also enriched for cytokine receptors and memory signature genes compared to naïve CD8 single positive thymocytes [34]. These changes correlate with a strong increase in histone H3K27ac on the promotors of genes upregulated in T_IM_ cells. This histone mark is associated with active transcription and active enhancers [74]. The strongest epigenetic changes between T_N_ and T_IM_ are found in enhancers. Further comparison of H3K27ac peaks between T_IM_ and T_TM_ shows that many of the epigenetic changes in T_IM_ cells compared to CD8 single positive thymocytes are similar to the changes observed between T_N_ and T_TM_ [34]. However, not all changes observed in T_TM_ also occur in T_IM_ [34]. This suggests that T_IM_ cells use similar epigenetic programs as conventional antigen-experienced T_TM_ cells, but also have T_IM_-specific changes in enhancer activity.

Interestingly, CD5^high^ T_N_ cells (CD44^−^CD62L^+^), which are more prone to differentiate toward T_VM_ compared to CD5^low^ T_N_ cells, already show upregulation of key T_AIM_ genes prior to memory differentiation [9]. CD5^high^ T_N_ cells also already show increased expression of cytokine genes associated with homeostatic cytokine responsiveness (*Il2rb*, *Eomes*, *Il4r*) compared to CD5^low^ T_N_ cells [9]. Furthermore, these cells show increased expression of gene clusters related to cell division and genes expressed in late effector and memory stages [67]. This upregulation of key T_AIM_ genes in T_AIM_ precursors seen in the peripheral CD5^high^ cells, is in line with increased protein levels of EOMES in thymic T_VM_- precursors [12]. These findings suggest that transcriptomic changes are not merely a result of having a more differentiated phenotype, but that they lie at the basis of antigen-inexperienced memory differentiation.

As mentioned before, T_AIM_ cells are a heterogeneous population, and this is also reflected in their transcriptome. Using the time-stamping technique Smith et al. isolated T_N_ (CD8^+^CD44^low^) and T_VM_ (CD8^+^CD44^high^) cells that developed during different time-points. Compared to T_VM_ cells produced during adulthood, T_VM_ cells produced early in life express more genes typically observed in short-term and late-effector CD8^+^ T cells after in vivo stimulation [17]. This also correlates with their chromatin landscape, with early-developed T_VM_ cells having increased accessibility at binding sites for transcription factors associated with effector differentiation. On the other hand, transcription factors involved in repression of effector differentiation have increased binding in chromatin more accessible in late-developed T_VM_ [17]. This suggests that the differential regulome of T_VM_ cells with different developmental origins affects their responsiveness to key differentiation transcription factors [17]. T_VM_ from aged mice have a more senescent phenotype that correlates with increased expression of anti-apoptotic transcripts including *Bcl2* and downregulation of exhaustion markers, contributing to the accumulation of T_VM_ over time [7]. Thus, the developmental origin of T_AIM_ cells determines their transcriptome and epigenome. The transcriptomic and epigenomic data for T_AIM_ cells described here has been acquired by bulk sequencing of T_AIM_ populations. Given the heterogeneity of the T_AIM_ pool, single-cell sequencing will be important to further understand how T_AIM_ differentiation relates to regulation of the transcriptome and epigenomes.

## Key epigenetic and transcriptional regulators in T_AIM_ differentiation

### EOMES

One of the key transcription factors expressed in T_AIM_ cells is EOMES [36]. EOMES is a member of the T-Box family of transcription factors and is upregulated during conventional memory differentiation/antigen exposure [75]. EOMES is more highly expressed in T_VM_ developed in the fetal stage compared to T_VM_ developed during adulthood, but in all T_VM_ subsets its expression is higher than in T_N_ cells [17]. EOMES is very important in T_AIM_ differentiation, as is shown by the strong reduction of T_AIM_ cells in thymus and spleen in *Eomes*-KO mice [15, 53]. This was further confirmed in a model with transgenic overexpression of *Eomes* in thymocytes, resulting in an increase in the percentage of T_IM_ cells [34].

EOMES is required for T_AIM_ differentiation at several levels. EOMES has been shown to bind directly to the promotor and internal regions of *Bcl2* and drive *Bcl2* expression (pro-survival protein), thereby giving memory cells a survival advantage compared to the strongly stimulated effector cells [76]. Besides driving pro-survival signals, EOMES is also involved in upregulation of CD122, which is part of the IL-15 receptor and upregulated in T_AIM_ cells [15, 29, 34, 37]. EOMES shows several interactions with other key regulators [34]. One potentially interesting interactor is RUNX3 because Runt motifs are slightly enriched in enhancer regions that have increased activity in T_IM_ vs T_N_ [34]. It has been suggested that EOMES is recruited toward regions where RUNX3 has bound [34]. In addition, BRG1, part of the SWI/SNIF chromatin remodeling complex, is critical to induce the EOMES-dependent program [34]. EOMES is thus a key transcriptional regulator in T_AIM_ cells that is embedded in a broader network of transcriptional and epigenetic regulators.

EOMES can be induced by several upstream factors including TCR stimulation and cytokines. IL-4 is one of the main drivers of EOMES expression in T_AIM_ cells. IL-4R knock-out (KO) mice show reduced EOMES levels in CD8^+^ T cells, including T_AIM_ cells [32], while in vivo injection with IL-4 leads to increased EOMES expression in splenic T_AIM_ cells [61]. In vitro, IL-4 is also sufficient to induce EOMES expression in CD8 single positive thymocytes [62]. Interestingly, this induction is stronger under submaximal TCR stimulation [62]. Also, in vivo submaximal TCR affinity results in the highest EOMES upregulation and drives central memory formation [76]. Besides IL-4, type 1 interferons (IFNs) can also induce EOMES expression as is shown by the reduced EOMES levels in T_AIM_ cells lacking the receptor or signaling pathway required for type 1 IFN pathway. Loss of the type 1 IFN pathway correlates with a reduction in the number of T_AIM_ cells. Mimicking type 1 IFN signaling by PolyI:C results in increased EOMES levels and increased T_AIM_ numbers in vivo [77]. Several signals involved in T_AIM_ differentiation are thus also involved in upregulation of the master T_AIM_ transcription factor EOMES.

### Epigenetic modulators

The epigenetic mechanisms involved in T_AIM_ differentiation are only starting to be unraveled, but several epigenetic factors have already been linked to T_AIM_ cells in different mouse models. The role of these epigenetic factors is mostly studied in conditional knock-out mouse models where the factor of interest is deleted in T cells during early T cell development. Among the epigenetic factors tested in this way is EZH2. EZH2 provides the catalytic part of the Polycomb repressive complex that is required for the generation of the repressive H3K27me3 mark [78–80]. Mice with conditional *Ezh2* KO in T cells have reduced H3K27me3 on the EZH2 targets, including the *Zbtb16* (PLZF) locus in iNKT cells. As a result, *Ezh2*-KO mice have aberrant iNKT differentiation and increased numbers of IL-4 producing iNKT cells, leading to increased numbers of T_AIM_ cells [81]. A similar mechanism is seen in mice lacking JARID2, a component of three lysine methyltransferase complexes that are involved in transcriptional repression, Polycomb repressive complex 2 (PRC2) that methylates histone 3 lysine 27 (H3K27), and the GLP-G9a and SETDB1 complexes that methylate H3K9. One of the targets of JARID2 is PLZF. Deletion of *Jarid2* results in reduced H3K9me3 on *Zbtb16* resulting in increased PLZF expression. This leads to an increase in IL-4 producing PLZF^high^ iNKT cells and as a result thereof more T_IM_ cells in the thymus [33]. Thus, deletion of EZH2 or JARID2 contributes to T_AIM_ differentiation at least in part by affecting thymic IL-4 production. Since EZH2 and JARID2 are major epigenetic regulators that affect the many aspects of the epigenome, it is likely that there are also other genes differentially expressed in these mouse models that contribute to T_AIM_ differentiation. T cell-specific deletion of the histone acetyltransferase CREB binding protein (CBP) also results in increased T_IM_ cells in a cell-extrinsic manner [14, 82]. This has been shown by a mixed bone marrow chimera experiment. When bone marrow cells from WT and *Crebbp*-KO were co-injected into the same mouse WT cells acquired the same phenotype as KO cells, indicating that CD8 cell-extrinsic factors are involved [14, 82].

In contrast to the above-described epigenetic regulators that affect T_AIM_ differentiation in a cell-extrinsic manner, other epigenetic regulators impact T_AIM_ differentiation cell intrinsically. The histone methyltransferase DOT1L is responsible for methylation of H3K79, which is associated with active transcription in gene bodies [83, 84]. Deletion of *Dot1L* in the T cell lineage results in a strong increase in the number of CD8^+^ T_AIM_ cells. This increase in T_AIM_ cells reflects a cell-intrinsic role of DOT1L as has been shown by mixed bone marrow chimeras [27]. When bone marrow cells from WT and *Dot1L*-KO were co-injected into the same recipient mouse, the WT cells remained mostly naïve, whereas *Dot1L*-KO cells still differentiated to T_AIM_ cells [27]. The onset of T_AIM_ differentiation in *Dot1L*-KO mice starts in the thymus where CD8 single positive thymocytes have increased expression of memory genes [27]. DOT1L-ablated T cells have aberrant expression of TCR signaling genes and reduced surface levels of the TCR complex [27]. This suggests that altered TCR signaling might be involved in T_AIM_ differentiation in *Dot1L*-KO mice. H3K79me2 in WT CD8^+^ T cells marks active genes and loss of DOT1L activity results in downregulation of a subset of H3K79-methylated genes, whereas other H3K79-methylated genes remain highly expressed [27]. What determines which genes are sensitive to loss of H3K79me2 is still unclear, but the downregulated H3K79me2 marked genes include genes encoding TCR, co-stimulatory components, and TCR signaling components [27]. *Dot1L*-KO CD8^+^ T cells also show a group of upregulated genes that lack H3K79me2 in WT cells, suggesting that DOT1L controls expression of other transcriptional regulators, in particular repressors that indirectly regulate these genes. EZH2 was identified as a candidate negative regulator that is controlled by DOT1L and that can account for at least part of the indirect effects [27]. Taken together, DOT1L normally prevents T_AIM_ differentiation by controlling TCR signaling and expression, and by regulating a network of other transcriptional and epigenetic regulators. How DOT1L maintains this network, through its catalytic activity toward H3K79 or also by other functions, remains to be determined [85–88]. It would also be interesting to understand what the role is of putative H3K79 methyl ‘reader’ proteins and DOT1L binding partners in T_AIM_ differentiation, since these factors affect DOT1L output and activity [83, 89]. Finally, epigenetic regulators that impact DOT1L activity, including the histone deacetylase HDAC1 and the H2B ubiquitination machinery [83, 90–92], are also candidate factors affecting T_AIM_ differentiation.

**Fig. 2:**
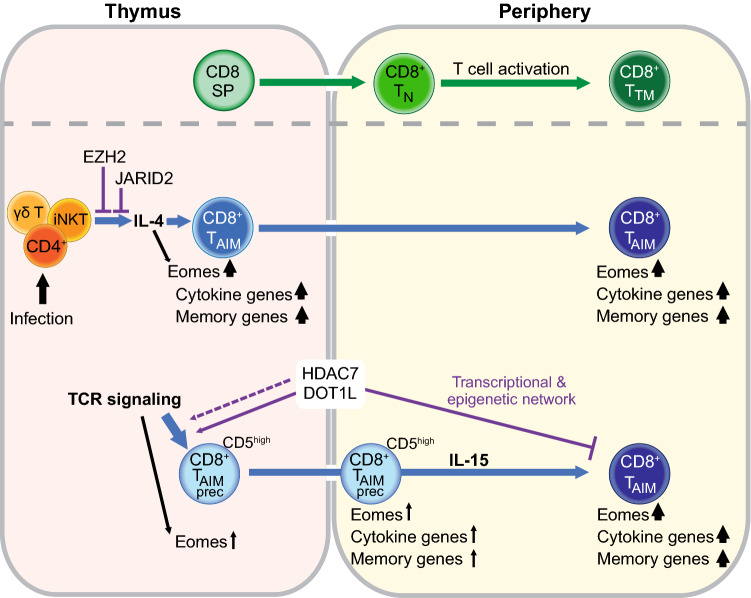
A model of T_AIM_ differentiation regulators. During conventional CD8^+^ T cell differentiation CD8 single positive (CD8 SP) thymocytes exit the thymus and enter the periphery as naïve (T_N_) CD8^+^ cells. Upon encountering a foreign antigen presented on the MHC class I complex of an antigen-presenting cells, T_N_ cells become activated and differentiate into short-lived effector and long-lived true memory (T_TM_) cells. T_TM_ cells provide a rapid immune response when re-activated, thereby ensuring an effective secondary immune response. In addition to the conventional T_TM_ cells, memory-phenotype cells also arise in an antigen-independent manner. The differentiation of the antigen-inexperienced memory T cells (T_AIM_) is guided by two main signals: cytokines and T cell receptor (TCR) signaling. Increased IL-4 levels in the thymus, either as a result of infection, or strain specific, or caused by deletion of the epigenetic modulator EZH2 or JARID2, induces T_AIM_ differentiation in CD8 SP thymocytes. These cells have increased expression of cytokine genes and memory genes, including the key transcription factor EOMES. EOMES expression can also be upregulated directly by IL-4. A specific level of heightened TCR signaling also affects EOMES expression and results in the upregulation of CD5 on naïve CD8 single positive thymocytes that are more prone to become T_AIM_ cells. Upon migration to the periphery, these CD5^high^ cells already express mildly increased levels of cytokine genes and memory genes. IL-15 signaling further drives these cells to become T_AIM_ cells. The histone modifiers DOT1L and HDAC7 prevent T_AIM_ differentiation by regulating transcriptional and epigenetic networks that keep cells in a naïve state. Furthermore, DOT1L and, possibly also, HDAC7 are involved in regulating TCR signaling

Another epigenetic modulator that has a central role in T_AIM_ differentiation is the histone deacetylase HDAC7 [93]. HDAC7 a class IIa HDAC that functions as a transcriptional repressor [94]. The concentration of HDAC7 in the nucleus is actively regulated during T cell development in the thymus [95]. Deletion of *Hdac7* results in reduced numbers of iNKT cells and a general reduction in the number of thymocytes, but in an increased percentage of T_IM_ cells [93, 95]. Using mixed bone marrow chimeras, it has been shown that this increase in T_IM_ cells is cell intrinsic [93]. However, it is not clear how deletion of *Hdac7* results in increased percentages of T_IM_ cells. HDAC7 itself has very minimal deacetylase activity but performs most of its catalytic function through interaction with the co-repressive N‐CoR/SMRT/HDAC3 complex [96–98]. In mature T cells, HDAC7 is continually phosphorylated resulting in translocation of HDAC7 from the nucleus to the cytosol [99]. In the thymus, HDAC7 is located in the nucleus during early thymocyte development and exported to the cytosol during positive selection [95]. The majority of the transcriptional changes observed during *Hdac7* deletion in thymocytes overlap with changes induced by positive selection [95]. This suggests that HDAC7 is a negative regulator of the downstream effects of TCR activation [95]. A possible mechanism for T_AIM_ differentiation in *Hdac7*-KO mice might thus involve increased TCR signaling. An overview of the role of epigenetic factors in T_AIM_ cells is outlined in Fig. 2.

Given the important role of epigenetic regulators in the execution of developmental transcriptional programs, it is likely that other epigenetic factors are also involved in T_AIM_ differentiation. Studying these regulators in conditional knock-out mouse models might prove difficult since other cells subsets are likely also affected in these mouse models. This is for example the case with EZH2. Upregulation of EZH2 target genes has been shown in *Dot1L*-KO T_AIM_ cells [27]. However, deletion of *Ezh2* in the T cell lineage has major effects on iNKT cell differentiation, thereby indirectly affecting T_AIM_ differentiation [81]. This strong effect of EZH2 on iNKT cell differentiation might mask the possible cell-intrinsic effects of *Ezh2* deletion in CD8^+^ T cells. More advanced conditional ablation models combined with detailed epigenomic and transcriptomic analysis, preferably on single-cell level, will help to further understand the epigenetic mechanisms behind T_AIM_ differentiation.

## T_AIM_ cells in humans

Most studies on T_AIM_ cells have been performed in mice. Recently some studies described T_AIM_-phenotype cells in humans. Their precise origin and function are not known, but they are present in umbilical cord blood and fetal spleen, suggesting their antigen-inexperienced nature, and in blood and liver from mature adults [9, 54, 100–102]. In young adults T_AIM_ cells constitute around 5% of the peripheral CD8^+^ T cell population and up to 15% in aged individuals [7, 103]. Human T_AIM_ cells are characterized by expression of the innate natural killer (NK) markers NKG2A or KIR and expression of the memory transcription factor EOMES [9, 101, 102]. Most putative human T_AIM_ cells in cord blood and in adult peripheral blood mononuclear cells have an effector memory re-expressing CD45RA (T_EMRA_) phenotype [9, 102, 103]. Similar to T_IM_ cells in mice, human putative T_AIM_ cells most likely also arise in response to IL-4. This has been studied in the context of chronic myeloid leukemia, a condition where iNKT activity, a major source for IL-4 production, is reduced. The first indication for IL-4 being important for human T_AIM_ differentiation is that the percentage of T_AIM_-phenotype cells is decreased in chronic myeloid leukemia, and that this decrease can be partially restored after complete remission of chronic myeloid leukemia [104]. Furthermore, stimulation with IL-4 in vitro results in an increased number of T_AIM_-phenotype CD8^+^ T cells [104]. The authors suggest that iNKT cells are the source of the T_AIM_-stimulating IL-4 since the level of EOMES on T_AIM_-phenotype CD8^+^ T cells correlates with the level of PLZF on iNKT cells [104]. IL-15 has also been suggested to play a role in the maintenance/differentiation of human T_AIM_-phenotype cells. KIR/NKG2A^+^EOMES^+^ T_AIM_-phenotype CD8^+^ T cells are preferentially expanded in HIV-infected patients [103]. Interestingly, HIV-infected untreated patients have increased concentrations of IL-15 in the lymph nodes and an increased population of bystander expanded memory-phenotype cells (defined as CD45RO^+^ CCR7^−^) [105]. The increase of T_AIM_-phenotype cells in HIV-infected patients might also be (partially) due to increased homeostatic proliferation as a result of reduced CD4^+^ T cell numbers, but this remains to be studied. These studies suggest that human T_AIM_-phenotype cells are physiologically regulated and can be expanded under certain conditions.

As with murine T_AIM_ cells, the function of human T_AIM_-phenotype cells has not been completely described. In vitro, the T_AIM_-phenotype KIR^+^/NKG2A^+^ CD8^+^ T cells rapidly produce IFNγ upon innate-like IL-12 + IL-18 stimulation [102, 104]. Furthermore, it has been suggested that T_AIM_-phenotype cells may contribute to control of the HIV viral reservoir [103]. However, further research is required to validate the antigen-naïve state of these putative human T_AIM_ cells and to further study their origin and functionality.

## Concluding remarks

Antigen-inexperienced memory cells form a substantial part of the CD8^+^ T cell pool. They represent a unique memory cell subset with its own functions, transcriptome and epigenome. The number and function of T_AIM_ cells increases with age and during infections. This indicates that T_AIM_ cell formation play a dynamic role in the immune system. The mechanisms and dynamics of T_AIM_ differentiation have only been studied in the past few years and it is likely that many conditions that impact T_AIM_ differentiation and functionality are yet to be discovered. For example, besides infections with helminths or trypanosomes, other infections that affect thymic IL-4 levels might also impact T_AIM_ differentiation.

The presence and heterogeneity of T_AIM_ cells shows that memory differentiation is not driven by a single process. Memory differentiation is guided by a complex network of different stimuli, involving TCR signaling and cytokines. Based on the conditions of their stimulation T_AIM_ cells have specific functional characteristics. On a transcriptomic level, T_AIM_ precursors already show increased expression of some key memory genes and fully differentiated T_AIM_ cells have their own unique transcriptome. These transcriptomic changes correlate with changes in the epigenome but further studies are required to fully understand how epigenetic regulators contribute to T_AIM_ differentiation by direct and indirect mechanisms.

In conclusion, CD8^+^ T_AIM_ cells are a unique and intriguing subset of memory cells that help us to understand the complexity of memory cell differentiation and how different types of memory cells each contribute in unique ways to the immune response.

## Data Availability

Not applicable. Not applicable.
